# Why Do Species Co-Occur? A Test of Alternative Hypotheses Describing Abiotic Differences in Sympatry versus Allopatry Using Spadefoot Toads

**DOI:** 10.1371/journal.pone.0032748

**Published:** 2012-03-30

**Authors:** Amanda J. Chunco, Todd Jobe, Karin S. Pfennig

**Affiliations:** 1 Department of Geography, University of North Carolina at Chapel Hill, Chapel Hill, North Carolina, United States of America; 2 Signal Innovations Group, Inc., Durham, North Carolina, United States of America; 3 Department of Biology, University of North Carolina at Chapel Hill, Chapel Hill, North Carolina, United States of America; Instituto de Higiene e Medicina Tropical, Portugal

## Abstract

Areas of co-occurrence between two species (sympatry) are often thought to arise in regions where abiotic conditions are conducive to both species and are therefore intermediate between regions where either species occurs alone (allopatry). Depending on historical factors or interactions between species, however, sympatry might not differ from allopatry, or, alternatively, sympatry might actually be more extreme in abiotic conditions relative to allopatry. Here, we evaluate these three hypothesized patterns for how sympatry compares to allopatry in abiotic conditions. We use two species of congeneric spadefoot toads, *Spea multiplicata* and *S. bombifrons*, as our study system. To test these hypotheses, we created ecological niche models (specifically using Maxent) for both species to create a map of the joint probability of occurrence of both species. Using the results of these models, we identified three types of locations: two where either species was predicted to occur alone (i.e., allopatry for *S. multiplicata* and allopatry for *S. bombifrons*) and one where both species were predicted to co-occur (i.e., sympatry). We then compared the abiotic environment between these three location types and found that sympatry was significantly hotter and drier than the allopatric regions. Thus, sympatry was not intermediate between the alternative allopatric sites. Instead, sympatry occurred at one extreme of the conditions occupied by both species. We hypothesize that biotic interactions in these extreme environments facilitate co-occurrence. Specifically, hybridization between *S. bombifrons* females and *S. multiplicata* males may facilitate co-occurrence by decreasing development time of tadpoles. Additionally, the presence of alternative food resources in more extreme conditions may preclude competitive exclusion of one species by the other. This work has implications for predicting how interacting species will respond to climate change, because species interactions may facilitate survival in extreme habitats.

## Introduction

What determines whether or not closely related species co-occur? Although the forces that govern species distributions have long been a focus of ecological study [1]–[5], ascertaining what factors set the boundaries between closely related species is of special interest for understanding the evolutionary and ecological implications of species interactions [6]–[8]. Factors driving individual species ranges, let alone those driving the overlap of related species' ranges, are complex, and include abiotic factors (e.g., temperature and precipitation), and biotic factors (e.g., resource availability, competition, and predation) [3]–[5], [9]–[11]. One way to evaluate why closely related species occur sympatrically in some regions but not others is to compare the abiotic conditions in sympatry versus allopatry. Doing so can provide insight into the degree to which abiotic factors, as opposed to biotic or historical factors, set the boundaries of co-occurrence between species. In particular, comparing sympatry and allopatry could support one of three hypothesized patterns of environmental variation underlying species co-occurrence. Because different types of interactions between the two species and their environment dictate each pattern, ascertaining how sympatry and allopatry differ lends insight into the types of factors driving co-occurrence of closely related species.

**Figure 1:**
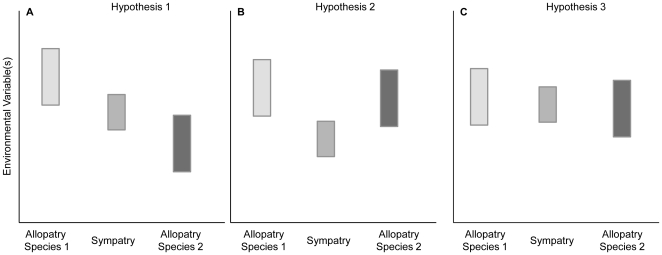
Alternative hypotheses for abiotic conditions underlying species distributions. A representation of three alternative patterns of environmental variation underlying sympatric and allopatric populations of two species across an environmental gradient. (a) Under hypothesis 1, species co-occur at intermediate environmental conditions where niches of the two species overlap. (b) Under hypothesis 2, biotic factors mediate co-occurrence such that species co-occur most commonly under extreme conditions. (c) Under hypothesis 3, sympatry and allopatry are governed primarily by dispersal ability, resulting in no environmental differences between sympatric and allopatric populations.

First, sympatry may be intermediate in abiotic environment compared to allopatry (hypothesis 1, Figure 1a). If abiotic factors are the primary drivers of species' ranges, then species should coexist wherever conditions fall within the fundamental niche of both species [4]. For example, if one species requires colder temperatures whereas the other requires warmer temperatures, then coexistence would occur at intermediate temperatures and only one species or the other will occur at more extreme temperatures (e.g. [12], [13]). More generally, this pattern is expected if range margins initially arise as a response to an underlying abiotic environmental gradient [14]–[16].

A second pattern that can emerge in comparing the abiotic conditions of sympatry and allopatry is that sympatry may occur in habitats that are more extreme than one of the allopatric regions (hypothesis 2, Figure 1b). In other words, sympatry may lie at one end of a continuum of environmental variable(s) as opposed to being intermediate between the two allopatric conditions. Such a pattern would emerge if *biotic* interactions mediated either, or both, species' responses to the underlying abiotic conditions in sympatry. Indeed, one species might facilitate the presence of a second species in extreme environments. This can arise with facultative mutualisms [17]–[19]. Similarly, hybridization may allow the colonization of extreme habitats via ‘genetic facilitation’ [20]. Moreover, an additional food resource may be present only in the extreme environment, or predators or parasites may be present that depress populations of one or both species, thereby precluding competitive exclusion of one species by the other [21]–[22]. Regardless of why sympatry occurs at one extreme of the abiotic environmental continuum, such a pattern would be primarily driven by biotic, rather than abiotic, factors.

Finally, the third pattern that could emerge in comparing abiotic conditions in sympatry and allopatry is that they do not differ (i.e. hypothesis 3, Figure 1c). Such a pattern would strongly suggest that dispersal limitation (either owing to aspects of the focal species' behaviour or physiology or due to physical barriers such as rivers or mountains) is the key factor limiting individual species' distributions within potential range boundaries [23]. Thus, sympatry and allopatry may arise owing to biogeographic history rather than an underlying environmental difference.

Distinguishing among the above patterns is important, because each hypothesis suggests how a different set of factors governs species ranges and regions of overlap between closely related species. Yet, evaluating how sympatry and allopatry differ regionally is often intractable, especially for wide ranging species. Generally, only a subset of environments within the species ranges and areas of overlap can be sampled or a small subset of variables measured. Consequently, comparing sympatry and allopatry based on field measures is often limited in the degree to which comparisons reflect range-wide patterns.

Here, we address these issues by combining niche modeling and environmental analysis to evaluate the above three alternative hypotheses for how regions of sympatry and allopatry might differ in abiotic conditions. We do so using two congeneric species of spadefoot toads, *Spea multiplicata* (Cope, 1863) and *S. bombifrons* (Cope, 1863) as our study system. In amphibians such as spadefoot toads, the abiotic environment is expected to be particularly influential on their ranges [24]. At the same time, complex biotic interactions between these species could influence their range dynamics. Indeed, as we explain in greater detail below, the tadpoles of *S. multiplicata* and *S. bombifrons* compete for resources in at least part of their range [25], [26]. Consequently, the abiotic environment can also indirectly affect the distribution of these species by governing the distribution of food resources for which they compete. Moreover, as we also describe below, the two species interbreed, and hybrid fitness is determined in part by the abiotic environment [27], [28]. Yet, whether such interactions scale up to affect regional patterns of sympatry and allopatry, as opposed to only affecting local distributions of the two species, is unclear. This system is therefore an excellent model for evaluating whether and how regions of sympatry and allopatry differ and can thereby provide insight into the relative importance of abiotic versus biotic forces in setting the regions of co-occurrence for closely related species.

## Methods

### Study system

*Spea multiplicata* and *S. bombifrons* inhabit arid regions of western North America [29]. Both species spend most of the year underground, and emerge to breed in ephemeral ponds that form after summer rains [30]. Because their offspring develop in ephemeral ponds, the ranges of both species should be highly sensitive to abiotic environmental conditions, such as temperature and rainfall, which affect pond duration.

**Figure 2:**
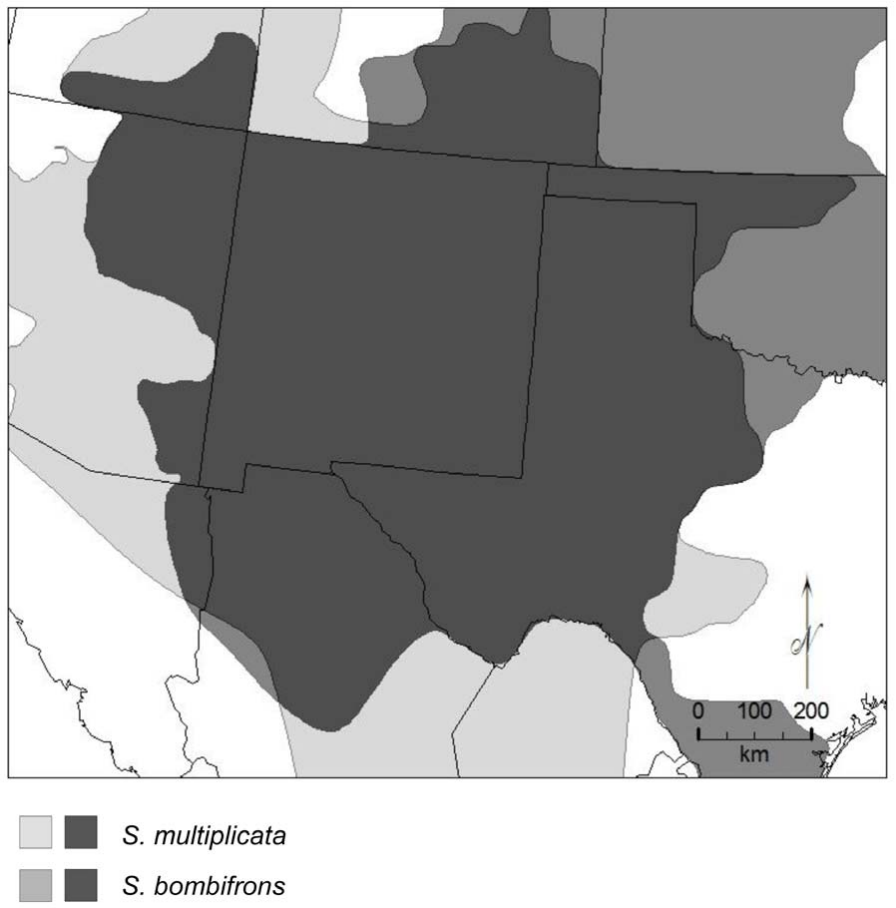
Range maps for *Spea multiplicata* **and** ***S. bombifrons*** **.** Range map showing the total range of both *S. multiplicata* and *S. bombifrons*.

Although range maps suggest a broad area of sympatry for *S. multiplicata* and *S. bombifrons* [29], [31] (Figure 2), whether these species actually co-occur in the same habitat through much of their range is unclear. Within southern Arizona and New Mexico, for example, these species show patterns of either co-occurrence or habitat segregation along an altitudinal gradient [21], [32].

Whether populations are actually sympatric or allopatric likely depends, at least in part, on interactions between species. Experimental work suggests that competition for food at the tadpole stage may, in part, drive patterns of species presence and absence [21]. Whereas adults of both species primarily feed on beetles and other small invertebrates [33], at the tadpole stage, *S. multiplicata* and *S. bombifr*ons both feed on anostracan shrimp and detritus. Where both species occur together, however, *S. bombifrons* tadpoles outcompete *S. multiplicata* tadpoles for one food resource, shrimp, whereas *S. multiplicata* outcompetes *S. bombifrons* for an alternate resource, detritus [34]. When only one food resource is abundant, only the species capable of specializing on that resource is found [21]. However, when both resources are abundant, both species are also usually present [21]. Thus, these species co-occur in habitats with alternative food resources available, but competitive exclusion appears to occur when one resource is insufficient [21]. Because abiotic factors can generally influence the availability of food resources, climate could directly affect the presence and absence of the spadefoot species via physiological constraints and also indirectly affect their presence/absence because of the effects of climate on resource availability.

Reproductive interactions may also contribute to patterns of species presence and absence. These species naturally hybridize, and hybridization has been observed in areas of sympatry [32], [35], [36]. Hybrid tadpoles feed on detritus and anostracan fairy shrimp, as do pure species tadpoles. However, hybrids appear competitively equivalent or even superior to pure species for both resources [37]. Nevertheless, the fitness consequences of hybridization are environmentally dependent and differ for the two species [27], [28]. In particular, in situations where ponds dry rapidly, hybridization is advantageous for *S. bombifrons* females, but not for *S. multiplicata* females, because hybrid offspring develop faster than pure species *S. bombifrons* offspring but slower than pure *S. multiplicata* offspring [27]. Because ponds frequently dry before tadpoles complete metamorphosis, fast development time is critical in fast drying ponds [38]. By contrast, when ponds are long lasting, hybridization is not favourable for either species because hybrid adults suffer reduced fertility [27]. Consequently, in environments where hybridization is deleterious for both species (i.e., long lasting ponds), hybridization could depress fitness and reduce population viability [39]. Alternatively, in environments where hybridization has a positive effect on *S. bombifrons*' development rate, the presence of *S. multiplicata* might actually facilitate the presence of *S. bombifrons*. Observations in the field have shown that hybridization is directional, with *S. bombifrons* females pairing with *S. multiplicata* males significantly more frequently than the reverse pairing [27]. Thus, in the spadefoots, the relative costs and benefits of hybridization in a given area––which are, in turn, determined by climate variables such a rainfall––may determine whether the two species co-occur as opposed to undergoing reproductive exclusion [10], [39].

### Niche modeling

To evaluate the alternative hypothesized patterns of abiotic conditions in sympatry versus allopatry (Figure 1), we first identified areas of likely co-occurrence between *S. multiplicata* and *S. bombifrons*. To do so, we independently modelled the presence of each species across their ranges using the niche modeling program Maxent set to the default values (ver. 3.3.2, [40]). Maxent was chosen because it demonstrates robust model performance compared to other modeling algorithms when presence-only data is available [41]. Moreover, because Maxent requires only presence data rather than presence-absence data [40], it was ideally suited for use with museum records to identify locations of species occurrence (see below). In doing so, our aims were to: 1) identify regions where *S. multiplicata* and *S. bombifrons* are predicted to co-occur, and 2) compare environmental conditions between regions where these species are and are not predicted to coexist.

Maxent uses species occurrence records and environmental data to build a predictive model of species distributions [40]. For species occurrence data, we compiled all *Spea bombifrons* and *S. multiplicata* records available from 29 museums throughout the United States, either via HerpNET (http:\\herpnet.org) or directly from the museums (n = 14,695; a list of the institutions that provided data is found in Table S1). Records with missing, incomplete, or inconsistent locality information or year of collection were excluded. Only one record from each unique location was used in the model, and duplicate locality records (i.e., all other records from the exact same geographic coordinates) were discarded. Each remaining record was then georeferenced, and the relative uncertainty was determined following the guidelines recommended by Chapman and Wieczorek [42]. Only museum records specific enough to identify the collection location to within one kilometer and those with a collection date between 1950 and 2000 were used in the model. This time frame corresponds with the years the climate data used to generate the WorldClim environmental layers were collected [43]. After selecting records that met the above criteria, 250 localities for *S. bombifrons* and 288 localities for *S. multiplicata* remained.

For environmental data, we initially considered 19 bioclimatic variables from WorldClim (www.worldclim.org ver. 1.4, [43]), and two hydrological variables from the U.S. Geological Survey's Hydro-1k dataset (http://eros.usgs.gov/#/Find_Data/Products_and_Data_Available/gtopo30/hydro). After removing highly correlated variables (see Methods S1), we were left with eight bioclimatic variables that were used in the final models.

Because variable selection can affect model results (e.g. [44]), we ran four separate models – using a different set of environmental variables in each model – for each species. Three models used different sets of abiotic environmental variables, whereas the fourth model used the model results for one species as the only variable in predicting the distribution of the other species (see Methods S2; Table S2). This Biotic Model was included because previous research has shown that including additional species in distribution models can improve model performance if those species interact in a biologically meaningful way [45]. After identifying the best performing model, we also performed a sensitivity analysis to evaluate the effects of the regularization multiplier (see Methods S2).

Maxent provides a logistic output where each grid cell value is the probability of occurrence relative to a randomly selected cell based on environmental suitability. The logistic values range from 0, signifying a low probability of occurrence (i.e. low habitat suitability), to 1, signifying a high probability of occurrence (i.e. high habitat suitability) [46]. We subsequently used these values to assign records from museum specimens to being from allopatric *S. bombifrons* regions, allopatric *S. multiplicata* regions or sympatric regions (see below).

### Comparing the environment in sympatry versus allopatry

We next compared the abiotic environment between regions of predicted sympatry and predicted allopatry as identified by the niche models. This allowed us to evaluate each of the three hypotheses outlined in the introduction (Figure 1), thereby providing important information about how the abiotic environment might mediate species coexistence. We were particularly interested in environmental differences between allopatric and sympatric sites in areas where species' ranges overlap (and thus areas with the potential for sympatry) rather than differences between species' range boundaries. We therefore used the localities of the museum records to construct a minimum convex polygon (i.e., a polygon described by points that fall at the outermost edge of the distribution) to define each species known range. Only museum record localities that fell within this minimum convex polygon were used in the environmental analysis. This provides a more appropriate and conservative comparison of environmental conditions between regions of sympatry and allopatry.

For each museum record that met the above criteria, we used the value of the Maxent logistic output from the best performing abiotic model (which was the Climate-Only model; see Results S1) to designate each specific site as either: 1) predicted sympatry, 2) predicted *S. multiplicata* in allopatry, 3) predicted *S. bombifrons* in allopatry, or 4) neither species present. We used the calculations below, where P(*m*) is the logistic value for *S. multiplicata*, and P(*b*) is the logistic value for *S. bombifrons*:

1. Probability of sympatry: 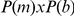

2. Probability of *S. multiplicata* in allopatry: 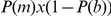

3. Probability of *S. bombifrons* in allopatry: 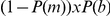

4. Probability of neither species present: 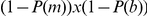

Each site was assigned to one of the four location types above based on which outcome was most probable (e.g. if a site had a probability of sympatry of 0.7 while the other three categories had a probability of 0.1, the site would be assigned to sympatry). All sites were assigned to the category with the highest probability, even when the difference between two categories was small. Although this method may not correctly assign every site, it is less arbitrary than using a binomial threshold approach (e.g. assigning sites that have a logistic value of above 0.5 for both models to sympatry, [47]), and it provides a conservative measure of sympatry.

**Figure 3:**
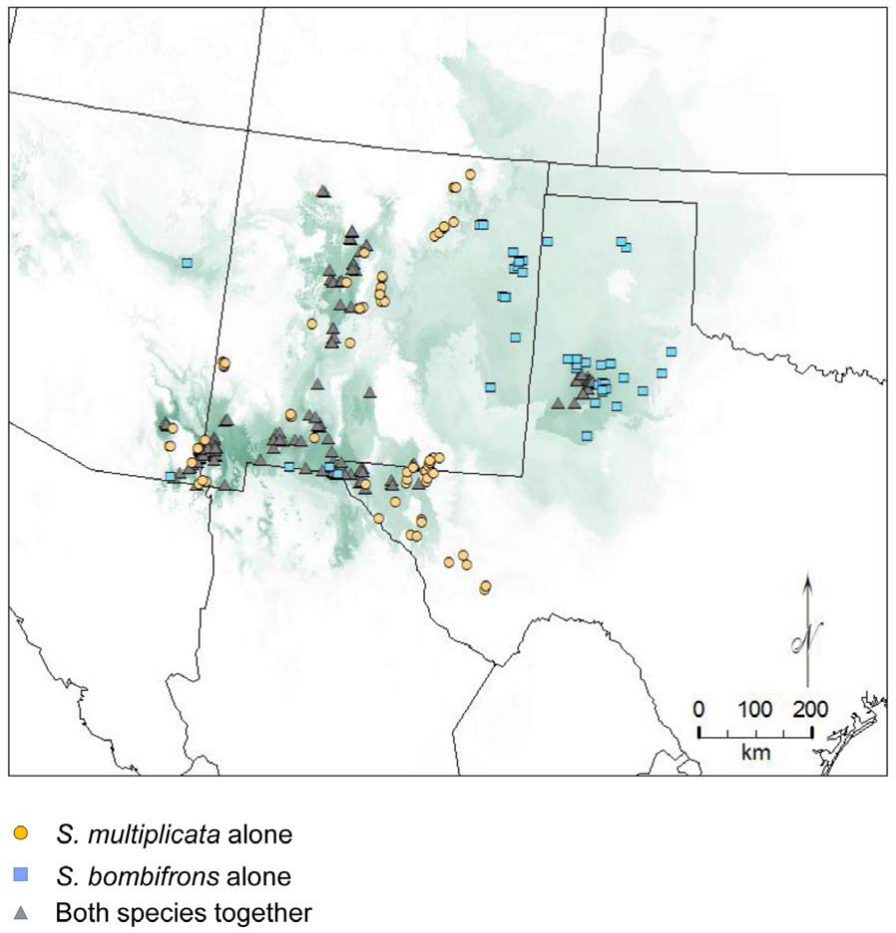
Range maps of predicted sympatry. Range maps of predicted sympatry between *Spea multiplicata* and *S. bombifrons*. The value for each 1 km sq pixel was calculated by multiplying the logistic value of both species, and values range from 0 (white) to 1 (dark green). Sites used in the environmental analysis are indicated by points. Specifically, blue squares represented collection locations for *S. bombifrons* that occurred in areas predicted to be allopatric for that species; orange circles represent collection locations for *S. multiplicata* records that were predicted to be allopatric for that species, whereas gray triangles represent collection locations for either species in areas predicted to be sympatric.

Because we were interested in the differences between regions predicted to be sympatric and those predicted to be allopatric, we restricted our analysis to sites where the niche model predicted either sympatry or allopatry. Thus, we removed locations where neither species was predicted to occur or where the locality was incorrectly assigned based on known occurrences from museum records (i.e. a locality where one species was collected that was from an area where the other species was predicted to be allopatric). *S. multiplicata* records that were identified by the model as *S. bombifrons* in allopatry were primarily found at the eastern edge of the range for *S. multiplicata*, while *S. bombifrons* records identified as *S. multiplicata* were primarily found at the western edge of the range for *S. bombifrons*. This suggests that these records might be found in sink habitats that are not favourable for long-term population persistence. Omission errors (i.e. incorrectly predicting a species' absence in areas where it is truly present) are expected in sink habitats [23]. Therefore, removing sites using the above criteria should not bias our results. Our final samples consisted of 120 *S. bombifrons* records (47 from predicted allopatry and 73 from predicted sympatry) and 170 *S. multiplicata* records (76 from predicted allopatry and 94 from predicted sympatry; the data from the 167 sympatric sites were combined for our comparisons of sympatry and allopatry below; Figure 3).

For each record designated as predicted sympatry or predicted allopatry, we extracted the value of each environmental variable used in the niche model from that location. We then compared all three location types: sympatry, allopatric *S. multiplicata*, and allopatric *S. bombifrons*. To evaluate the composite environmental differences between allopatry and sympatry, we performed a principal components analysis. We retained all principal components with an eigenvalue greater than 1 and used ANOVA to discriminate whether the location types were significantly different in terms of each retained principal component.

Finally, to further visualize the range of conditions experienced by each group, we used box-and-whisker plots to show each environmental variable individually in addition to the PCA. All statistical calculations were preformed in R v 2.10.1 [48].

## Results

In generating the alternative niche models, we found that, although each model used a different subset of environmental variables, all four models showed similar areas of predicted occurrence (Figure S1, Figure S2, Figure S3). The three abiotic models showed very similar regions of moderate suitability, and these models reveal a substantially smaller region of sympatry than would be assumed based on range maps alone (Figure S1, Figure S2, Figure S3). In contrast, the Biotic Model showed a larger area of moderate habitat suitability than any of the abiotic models (Figure S1, Figure S2, Figure S3). All maps shown are the average of the ten replicate runs for each model.

The abiotic model with the best performance in terms of AUC included only the eight climate variables (i.e, the Climate-Only Model, Table S3), and the results of this model were used to evaluate sympatry and allopatry (Figure 3). The sensitivity analysis showed the effect of the regularization multiplier (Figure S4, Table S4). Although the regularization multiplier influences the extent of highly suitable habitat predicted by the model, the major areas of sympatry are the same among all models. Because Philips and Dudik [46] suggest that the default values of Maxent are appropriate for a variety of conditions, we used the default regularization multiplier value of 1.0 for our analysis of sympatry and allopatry.

As evidence that these models were good descriptors of sympatry and allopatry, nearest neighbour distances to the closest record of the other species were significantly closer in predicted sympatry (mean distance = 7.02 km) than predicted allopatry (mean = 20.33 km) for *S. bombifrons* (t_118_ = 3.785, p<0.001,) and, likewise, for *S. multiplicata* (mean in sympatry = 12.63 km, mean in allopatry = 51.52 km, t_168_ = 7.659, p<0.001). Moreover, most sites showed a substantial difference between the highest logistic value (i.e. the value used to assign a site to a particular geographic category) and the second highest value. The average logistic value (+/−SD) used to assign a site was 0.46 (+/− 0.13), whereas the average second highest logistic value (i.e. the next most likely geographic category) was 0.28 (+/− 0.06). Thus, assignments to predicted regions of sympatry or allopatry were not based on marginal differences in likelihood: most sites were unequivocally assigned to a particular geographic category.

**Figure 4:**
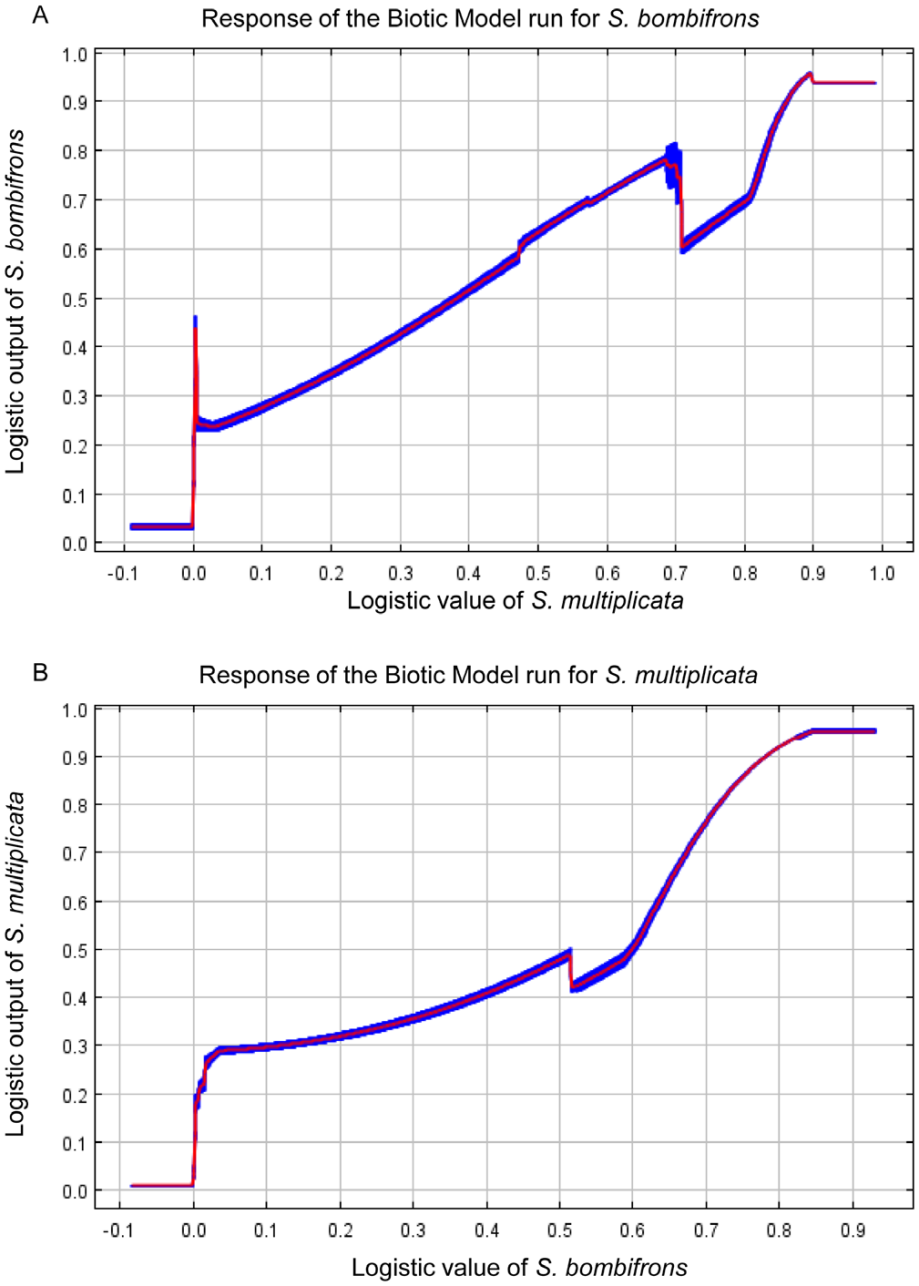
Maxent response curves for the Biotic Model. The response curves of the Biotic Model for (a) *S. bombifrons* and (b) *S. multiplicata*. These curves show how the logistic output changes along an ‘environmental gradient’. Here, the environmental gradient is the predicted output of the other species used to create the Biotic Model. The red line shows the average of the 10 replicate runs, while the blue bands shows +/− one standard deviation. At low logistic values for one species, the other species has a low logistic value as well. Both species thus show a similar response to the environment (i.e. environments good for one species tend to be good for the other).

The Biotic Model evaluated whether the predicted presence of one species could predict the presence of the other species. Examining the response curves for the Biotic Model shows how the logistic output changes along the environmental gradient (where the “environmental gradient” is the predicted output of the other species). We found that the Biotic Model performed reasonably well, and that the relationship was roughly positively linear (Figure 4). At low logistic values for one species, the other has a low logistic value as well, so both species show a roughly similar response to the environment (Figure 4). These results therefore indicate that the habitat requirements between the two species are similar. Nevertheless, this Biotic Model is the poorest performing model of the four that we considered. Moreover, the predicted areas of species presence were greater than in any of the other models, suggesting some over-prediction. This indicates that, although the requirements of *S. multiplicata* and *S. bombifrons* are similar, there are important differences between them in how they respond to the environment. Thus, models that include climate variables are still a better approach than using only the presence of one species to predict the distribution of the other.

**Figure 5:**
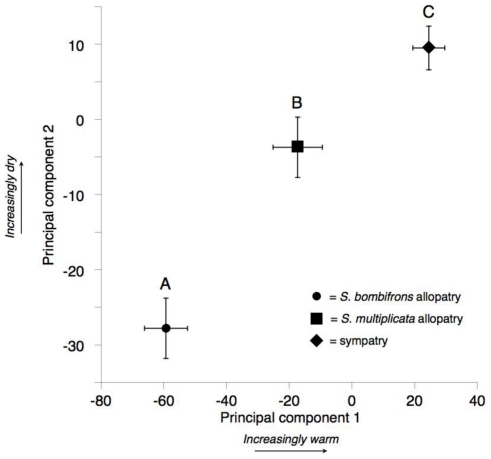
Principal components of the abiotic environment. Means (+/− s.e.) for the first two principal components describing variation in the eight environmental variables used to build ecological niche models. Different letters indicate significantly different means; each group (*S. multiplicata* in allopatry, *S. bombifrons* in allopatry, and sympatry), is significantly different from the other two.

To evaluate the combined differences in abiotic variables between sympatry and allopatry, we first used a principal component analysis (PCA). We retained the first two principal components (PCs), which, together, explained 87.5% of the variance. The loadings of each environmental variable on the first two principal components are shown in Table 1. When we contrasted these principal components between sympatry, allopatry for *S. bombifrons*, and allopatry for *S. multiplicata* using an ANOVA, we found a significant effect of region on both PC scores (PC 1: F(2, 287) = 35.522, p<0.001; PC 2: F(2, 287) = 21.678, p<0.001). A Tukey HSD test further revealed that all groups were significantly different from each other for both PC 1 and PC 2 at p<0.02 for all group comparisons. Moreover, the mean sympatric score for both principal components was greater than the mean scores for either allopatric region, indicating that sympatry occurs in extreme, rather than intermediate, habitats relative to allopatric regions (Figure 5). Because temperature in the driest quarter and precipitation in the warmest quarter loaded most strongly on PC 1 and PC 2 respectively (Table 1), our results indicate that sympatry is warmer and drier than either allopatric region (Figure 5). This pattern of sympatry being extreme in abiotic conditions, rather than intermediate relative to allopatric regions, is most consistent with hypothesis 2 in Figure 1.

**Table 1:** Loadings on the first two principal components for the eight environmental variables used in the Maxent model.

	PCA 1	PCA2
Mean Diurnal Range in Temperature	*	0.143
Maximum Temperature of Warmest Month	0.160	−0.176
Annual Range in Temperature	*	0.279
Mean Temperature of Driest Quarter	0.914	−0.169
Mean Temperature of Coldest Quarter	0.243	−0.158
Precipitation of Driest Quarter	*	*
Precipitation of Warmest Quarter	−0.214	−0.896
Precipitation of Coldest Quarter	0.105	−0.177

Loadings near 0 (i.e., −0.1 to 0.1) are denoted with an “*”.

**Figure 6:**
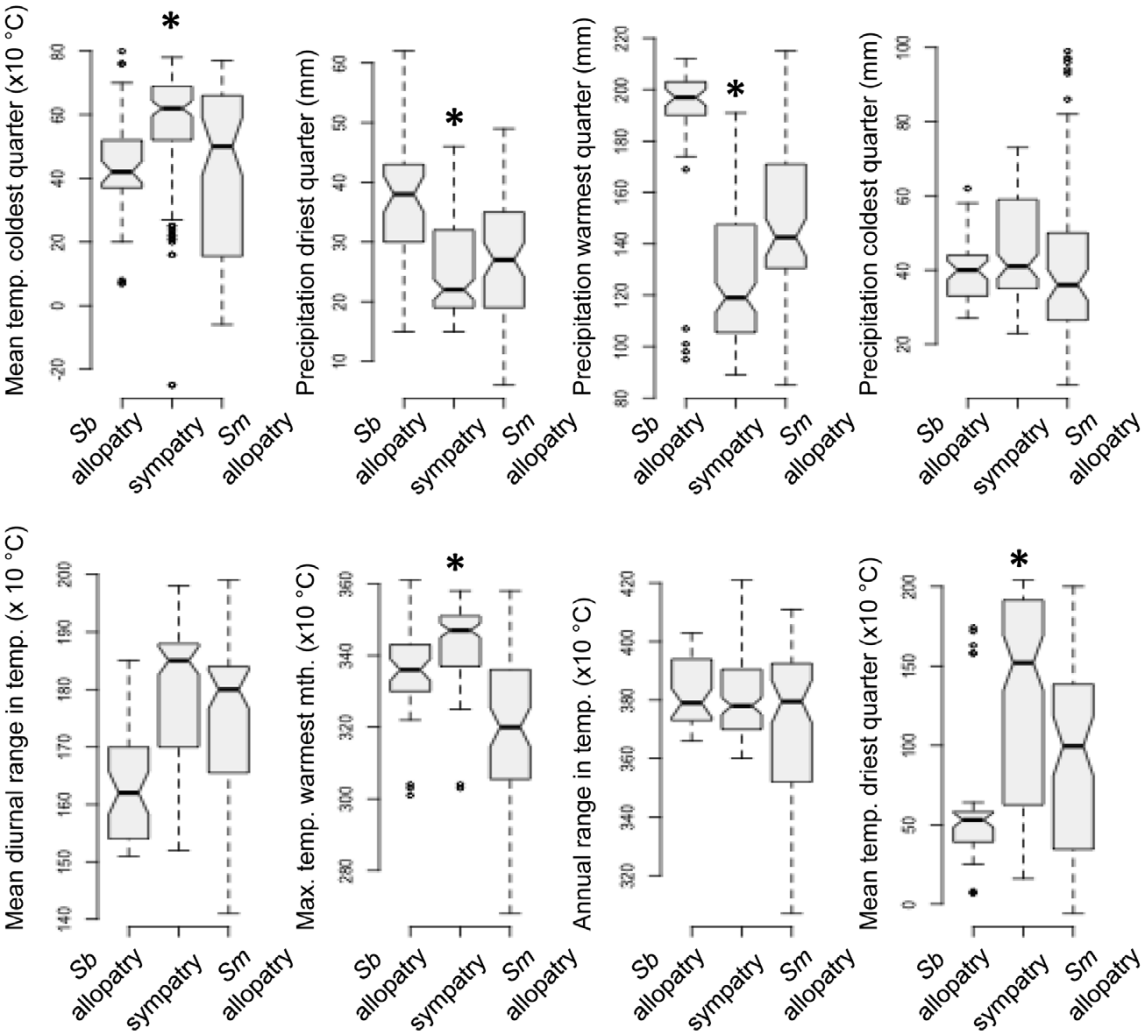
Environmental variation between sympatry and allopatry. Box-and-whisker plots showing environmental space occupied by predicted allopatric populations of *S. bombifrons* (abbreviated “*Sb* allopatry”), predicted allopatric populations of *S. multiplicata* (abbreviated “*Sm* allopatry”), and predicted sympatric populations of both species for each environmental variable used in the Maxent models. Non-overlapping notches are roughly equivalent to 95% confidence intervals, and therefore provide strong evidence that the medians differ [48], [58]. An “*” above the sympatry box indicates variables for which sympatric sites are significantly hotter or drier than both allopatric sites.

That sympatry occurs in regions at an extreme in abiotic conditions relative to allopatric regions is further emphasized when evaluating the individual variables used in the PCA. Allopatric *S. bombifrons* sites are wetter, cooler, and less variable in diurnal temperature range than allopatric *S. multiplicata* sites (Figure 6). Contrary to hypothesis 1, sympatry was not intermediate for any of the individual environmental variables. Instead, most variables (including: mean temperature of the coldest quarter, precipitation of the driest quarter, precipitation of the warmest quarter, maximum temperature of the warmest month, and mean temperature of the driest quarter) show median values for sympatry that are at an extreme relative to either species in allopatry (Figure 6). Generally, sympatry tended to be hotter and drier than allopatric sites for either species. Of all the environmental variables, only precipitation of the coldest quarter and annual range in temperature had median values that were similar between both species in sympatry and allopatry (Figure 6). Thus, contrary to commonly held views of sympatry and allopatry, sympatric regions between *S. bombifrons* and *S. multiplicata* were at an extreme of the abiotic environment relative to regions of allopatry.

### Summary

We used spadefoot toads (*Spea* spp.) as a case study for examining the factors potentially contributing to patterns of distribution and co-occurrence. We did so by constructing an independent predictive map of the entire range for *S. multiplicata* and *S. bombifrons*. We then contrasted the abiotic environment in areas where the two species were likely to co-occur (predicted sympatry) versus areas where each species was likely to occur alone (predicted allopatry).

The resulting niche models showed that sympatry is geographically interspersed within regions of allopatry for both species (Figure 3). Thus, we do not find a gradient of predominantly *S. bombifrons* habitat, then sympatry, then *S. multiplicata* habitat along a north-south axis, as might be expected in a contact zone between two species that differ latitudinally in distribution. Moreover, when we compared abiotic conditions between predicted sympatric sites and predicted allopatric sites, we found striking differences. Contrary to the common expectation that sympatric sites will be intermediate between allopatric sites in environmental variables [14], [16]; hypothesis 1 from Figure 1), we instead found sympatry to generally occupy more extreme ends of the distributions for the abiotic variables used in our study (Figures 5, 6). Specifically, sympatric sites tend to be hotter and drier than allopatric sites for either species. Allopatric *S. bombifrons* sites are substantially cooler and wetter than sympatric sites, whereas allopatric *S. multiplicata* sites appear more similar to sympatry than to allopatric *S. bombifrons* sites (Figures 5, 6).

## Discussion

Although a few studies have previously used Maxent to study patterns of species co-occurrence (e.g. [49], [50]), these studies have primarily relied on a threshold approach (i.e. using a predetermined cut-off in logistic value to identify presence and absence). The specific areas of predicted presence with the threshold approach depend, however, on the specific threshold chosen [40]. Instead, using the joint probability to highlight areas of co-occurrence, as we did here, provides several advantages over a threshold approach. First, joint probability is less arbitrary than choosing a binomial threshold to predict sympatry. Second, this approach provides a continuous measure of the probability of sympatry across the region of interest rather than a binomial measure of presence or absence (e.g. Figure 2). The continuous measure provides more information about the relative likelihood of co-occurrence, and is therefore potentially more useful for targeting field surveys. It is important to note that while Maxent highlights suitable environments for occurrence, suitable environment does not necessarily guarantee occurrence. Thus, some over-prediction is likely. However, given that these models perform well, and that multiple modeling approaches highlight similar areas of habitat suitability, these results will provide useful data for identifying new populations in the field.

The presence of sympatry in extreme rather than intermediate habitats (relative to allopatry) suggests that simple responses to the abiotic environment are not the primary factors mediating co-occurrence between *S. bombifrons* and *S. multiplicata*. Instead, our results suggest that biotic interactions within these extreme environments may be important in driving co-occurrence between *S. bombifrons* and *S. multiplicata*. In this case, the most likely biotic factor that could explain our results is hybridization. The two species are known to hybridize naturally [27], [32], [35], and *S. bombifrons* benefits by hybridizing in rapidly drying pools [28]. Specifically, hybrids between *S. bombifrons* and *S. multiplicata* develop faster than pure species *S. bombifrons* [27]. Beneficial hybridization may foster co-occurrence in drier, warmer habitats if hybridization allows *S. bombifrons* to maintain populations in habitats where pure species would otherwise be unable to persist. That the environment of sympatry tends to be more similar to *S. multiplicata* in allopatry than to *S. bombifrons* in allopatry is further consistent with the idea that hybridization with *S. multiplicata* facilitates the presence of *S. bombifrons* in warm, dry habitats that are dissimilar to sites found for allopatric *S. bombifrons*. Indeed, genetic data suggests that *S. bombifrons* expanded its range out of the Great Plains into the Southwestern USA [51]; our results suggest that hybridization may have fuelled this expansion into novel habitats.

An alternative (albeit not mutually exclusive) explanation for our results is that sympatry occurs where resources are available that foster coexistence. Indeed, the pattern of sympatry occurring in more extreme habitats could arise if such habitats foster the presence of alternative resources that minimize competition between ecologically similar species. Because *S. multiplicata* and *S. bombifrons* specialize on different resources (detritus and shrimp respectively) as tadpoles where they co-occur [25], [34], sympatric regions might occur in a more extreme environment than one of the allopatric regions if both resources occur in those environments. For example, anostracan fairy shrimp on which the tadpoles feed are potentially more abundant in hotter, drier habitats [52]. If such habitats contain sufficient detritus, then the presence of both resources might preclude competitive exclusion of one species by the other. Consistent with this notion, field observations have shown that local co-occurrence of both *Spea* species occurs only where both shrimp and detritus are sufficiently abundant to permit co-existence rather than competitive exclusion with southern Arizona and New Mexico [21].

Whether hybridization or the presence of sufficient resources (or both factors in combination) fosters sympatry between *S. multiplicata* and *S. bombifrons* will require further investigation. Because Maxent uses species locality data in predicting ranges, any factors that limit species ranges are indirectly incorporated into the model. Therefore, although we used climate to predict the ranges of both species, any factors (such as resources) that are themselves tightly correlated with climate will be indirectly included in the model. Using our models, we therefore cannot determine whether climate *directly* mediates co-occurrence (with hybridization facilitating *S. bombifrons*' persistence in hotter, drier habitats) or whether climate *indirectly* mediates coexistence via its effects on resources that mediate competition. Nevertheless, the results of this study can be used to guide empirical work testing specific predictions that arise from environmental comparisons of sympatry and allopatry. More generally, this approach of blending niche modeling with analysis of environmental data can guide greater insight into the factors that affect species co-occurrence.

These results also have implications for considering how the environment mediates species interactions when predicting responses to climate change. Many current models of future species' distributions assume that range limits are driven primarily by climate. Thus, although the potential for novel communities to form under future climate regimes has been well-documented [53], [54], few studies have explicitly considered how species interactions will be influenced by the environment (but see [55]). Here, we show that sympatric sites occur in regions that are hotter and drier than allopatric sites of either *S. bombifrons* or *S. multiplicata*. Because the U.S. southwest, where these *Spea spp*. are found, is predicted to become hotter and drier as climate change progresses [56], sympatry may become more extensive if the environment becomes more suitable for species co-occurrence. Indeed, if hybridization fosters *S. bombifrons*' ability to invade such habitat, such changes could alter both patterns of co-occurrence and genetic exchange between the two species. Thus, climate change may alter both evolutionary processes and ecological processes in these species.

Furthermore, these kinds of non-intuitive relationships between the environment and species interactions may explain in part why many studies of recent range changes have shown higher than expected variation in the response to climate change, with as many as ∼40% of species showing either no changes in range limits or a change in the opposite direction than predicted [57]. Therefore, future studies should more explicitly consider how the environment mediates species interactions when predicting responses to climate change.

## Supporting Information

Methods S1 **A description of the process used to select environmental layers for niche model construction.**
(DOCX)

Methods S2 **A description of: 1) each of the four niche models run, 2) the methodology for identifying areas of sympatry, and 3) the sensitivity analysis.**
(DOCX)

Results S1 **A description of the result of each of the four niche models run and the results of the sensitivity analysis.**
(DOCX)

Figure S1 **Maxent models for** ***S. bombifrons*** **.** Map of *S. bombifrons* under all 4 models. Each pixel visually represents the logistic value for *S. bombifrons* for each of the 4 models, where values range from 0 (shown in white) to 1 (shown in dark blue). The four models are: A) the Full Abiotic Model, B) the Climate-Only Model, C) the Summer Environment & Seasonality Model, and D) the Biotic Model.
(TIF)

Figure S2 **Maxent models for** ***S. multiplicata*** **.** Map of *S. multiplicata* under all 4 models. Each pixel visually represents the logistic value for *S. multiplicata* for each of the 4 models, where values range from 0 (shown in white) to 1 (shown in dark orange). The four models are: A) the Full Abiotic Model, B) the Climate-Only Model, C) the Summer Environment & Seasonality Model, and D) the Biotic Model.
(TIF)

Figure S3 **Maxent models of sympatry.** Map of predicted sympatry under all 4 models. The value for each pixel was calculated by multiplying the logistic value of both species for each model, and values range from 0 (shown in white) to 1 (shown in dark green). The four models are: A) the Full Abiotic Model, B) the Climate-Only Model, C) the Summer Environment & Seasonality Model, and D) the Biotic Model.
(TIF)

Figure S4 **Sensitivity analysis.** Maxent model results at four different regularization multipliers: A) 0.1, B) 0.5, C) 2.0, and D) 5.0. Each of these models was run based on the same environmental variables included in the Climate-Only model. Other than the regularization multiplier, all other values were held at default levels. Each map shows the average of 10 replicate runs.
(TIF)

Table S1 **The following institutions provided locality data used in the ecological niche models.**
(DOCX)

Table S2 **Environmental variables used in each Maxent model.**
(DOCX)

Table S3 **Mean and standard deviation for each of the four niche models run.**
(DOCX)

Table S4 **Mean and standard deviation for each of the four niche models run for the sensitivity analysis.**
(DOCX)
