# Supplementary material for: Why Do Species Co-Occur? A Test of Alternative Hypotheses Describing Abiotic Differences in Sympatry versus Allopatry Using Spadefoot Toads
Source: PLoS One. 2012 Mar 30;7(3):e32748. doi: 10.1371/journal.pone.0032748 (PMC3316550; doi:10.1371/journal.pone.0032748)
Supplement: Methods S1 — A description of the process used to select environmental layers for niche model construction. (DOCX) [file pone.0032748.s001.docx]

**Methods S1**

***Selecting Environmental Layers***

In evaluating which environmental data to use for the Maxent modeling, we initially considered 19 bioclimatic variables from WorldClim ([www.worldclim.org](http://www.worldclim.org) ver. 1.4; [1]) and two hydrology layers from the U.S. Geological Survey’s Hydro-1k dataset (<http://eros.usgs.gov/#/Find_Data/Products_and_Data_Available/gtopo30/hydro>). Because both *S. multiplicata* and *S. bombifrons* are ectotherms that rely on ephemeral ponds for reproduction, temperature and precipitation should be major drivers of their ranges. Furthermore, pond duration, which is driven in large part by temperature, rainfall, and hydrology, is likely particularly important to species presence in a given area because both species require ponds that persist long enough for tadpoles to achieve metamorphosis. Although *Spea spp*. develop remarkably rapidly [2,3] ponds frequently dry before tadpoles reach metamorphosis [4]. Thus, bioclimatic variables related to temperature and precipitation and hydrology variables (specifically the slope and wetness variables from the USGS hydrology database) are biologically relevant for *Spea spp*.

To reduce multicollinearity in the 19 original bioclimatic variables, we calculated the Pearson correlation coefficient (r) between each pair of variables using data from the location of each species occurrence. For each pair of highly correlated variables (r>.75), we selected only the single variable that was most biologically relevant for spadefoot toads. After this procedure, eight bioclimatic variables remained (Table S2). All environmental variables had a resolution of 1km x 1km.

**REFERENCES**

1. Hijmans RJ, Cameron SE, Parra JL, Jones PG, Jarvis A. (2005) Very high resolution interpolated climate surfaces for global land areas. Int J Climatol 25(15): 1965-1978. 10.1002/joc.1276.

2. Pfennig KS, Simovich MA. (2002) Differential selection to avoid hybridization in two toad species. Evolution 56(9): 1840-1848.

3. Bragg AN. (1965) Gnomes of the night: The spadefoot toads. Philadelphia, Pennsylvania: University of Pennsylvania Press.

4. Pfennig DW. (1992) Polyphenism in spadefoot toad tadpoles as a locally adjusted evolutionarily stable strategy. Evolution 46(5): 1408-1420.
