# Supplementary material for: Why Do Species Co-Occur? A Test of Alternative Hypotheses Describing Abiotic Differences in Sympatry versus Allopatry Using Spadefoot Toads
Source: PLoS One. 2012 Mar 30;7(3):e32748. doi: 10.1371/journal.pone.0032748 (PMC3316550; doi:10.1371/journal.pone.0032748)
Supplement: Methods S2 — A description of: 1) each of the four niche models run, 2) the methodology for identifying areas of sympatry, and 3) the sensitivity analysis. (DOCX) [file pone.0032748.s002.docx]

**Methods S2**

***Niche Modeling and Model Selection***

We ran four independent Maxent models for both *S. multiplicata* and *S. bombifrons* to identify the best set of variables to include in the final model and to determine how susceptible results were to variable selection. The first three models incorporated only abitoic variables: 1) the Full Abiotic Model included all ten climate and hydrology variables; 2) the Climate-Only Model used only climate variables; and 3) the Summer Environment and Seasonality model included hydrological variables, climate variables measuring seasonality, and climate variables from the warmest and wettest periods of the year when both species are active (Table S2). Because spadefoots aestivate underground for much of the year [1], we ran this model to see if excluding climate variables from cold, dry periods when animals are not exposed to the environment would improve model performance. Finally, because these species interact, we ran a Biotic Model using only the predicted presence of the other species based on the output of the Climate-Only Model (as this model had the highest AUC of the three abiotic models, Table S3). The Biotic Model provided further evidence on the degree of niche overlap between the species. In particular, if this model accurately predicts the location of each species, then the habitat requirements of the two species are similar. These four models each capture biologically relevant aspects of the species’ environment and were chosen to represent the suite of variables that might be important in driving distributions.

Each model was replicated 10 times using the cross-validation function in Maxent to partition the data into replicate folds, with each fold being used in turn to test the model [2]. The best performing model (as indicated by the highest area under the curve AUC of the receiver operatic characteristic plot (ROC, [3,4]; Table S3) included all eight bioclimatic variables and no hydrology data. We therefore used this model to identify regions of predicted sympatry and allopatry.

To determine where co-existence is most likely for each model run, we overlaid the Maxent logistic output for both species in ArcGIS 9.3 using an Albers Equal Area projection. We then multiplied the logistic output of both models (i.e. *S. bombifrons* and *S. multiplicata*) to determine the joint probability for each 1km sq grid; this joint probability provides a quantitative comparison of the likelihood of co-occurrence across the entire region considered.

Finally, we also performed a sensitivity analysis to evaluate the effect of the regularization multiplier on both AUC and the extent of predicted highly suitable habitat. The regularization multiplier is used to constrain the model, such that smaller values of the regularization multiplier give a more constrained and local distribution than larger values [5,6]. We therefore adjusted the regularization multiplier in each of four additional models (to values of 0.1, 0.5, 2, and 5 respectively). Note that the default value of the regularization multiplier was used in the models above and is 1.0. In all of these additional models, we used the same eight environmental layers as used in the Climate-Only model.

**REFERENCES**

1. Bragg AN. (1965) Gnomes of the night: The spadefoot toads. Philadelphia, Pennsylvania: University of Pennsylvania Press.

2. Phillips SJ, Dudik M. (2008) Modeling of species distributions with Maxent: New extensions and a comprehensive evaluation. Ecography 31(2): 161-175. 10.1111/j.0906-7590.2008.5203.x.

3. Pearce J, Ferrier S. (2000) Evaluating the predictive performance of habitat models developed using logistic regression. Ecol Model 133(3): 225-245. DOI: 10.1016/S0304-3800(00)00322-7.

4. Elith J, Graham CH, Anderson RP, Dudík M, Ferrier S, et al. (2006) Novel methods improve prediction of species’ distributions from occurrence data. Ecography 29(2): 129-151. 10.1111/j.2006.0906-7590.04596.x.

5. Elith J, Phillips SJ, Hastie T, Dudík M, Chee YE, et al. (2011) - A statistical explanation of MaxEnt for ecologists. Diversity and Distributions 17(1): 43-57.

6. Warren DL, Seifert SN. (2011) Ecological niche modeling in Maxent: The importance of model complexity and the performance of model selection criteria. Ecol Appl 21(2): 335-342. 10.1890/10-1171.1.
