# Supplementary material for: Why Do Species Co-Occur? A Test of Alternative Hypotheses Describing Abiotic Differences in Sympatry versus Allopatry Using Spadefoot Toads
Source: PLoS One. 2012 Mar 30;7(3):e32748. doi: 10.1371/journal.pone.0032748 (PMC3316550; doi:10.1371/journal.pone.0032748)
Supplement: Results S1 — A description of the result of each of the four niche models run and the results of the sensitivity analysis. (DOCX) [file pone.0032748.s003.docx]

**Results S1**

All four models for both species performed well, with AUCs of at least 0.736 (Table S3). The three abiotic models (Full Abiotic Model, Climate-Only Model, and the Summer Environment and Seasonality Model) had very similar results in terms of AUC. The Summer Environment and Seasonality Model, with only five variables, is thus sufficient to capture enough relevant environmental information to produce a map that performs very well in terms of AUC (Table S3). Adding additional information about the winter climate in the Full Abiotic Model improves model performance, whereas including all the climate data and no hydrological variables (i.e. the Climate-Only Model) performs best overall. Thus, capturing the full range of climatic conditions experienced by these species throughout the year is most useful in modeling their distribution.

The sensitivity analysis revealed that the value of the regularization multiplier did affect the extent of predicted habitat suitability for our focal species. Specifically, at low values, the models predict a more limited distribution that is more highly localized around the species locality records. As the multiplier increases, the area of high habitat suitability increases (Figure S4). Furthermore, the AUC values for these four models were statistically similar (Table S4).
