# Supplementary figures and images for: Why Do Species Co-Occur? A Test of Alternative Hypotheses Describing Abiotic Differences in Sympatry versus Allopatry Using Spadefoot Toads
Source: PLoS One. 2012 Mar 30;7(3):e32748. doi: 10.1371/journal.pone.0032748 (PMC3316550; doi:10.1371/journal.pone.0032748)

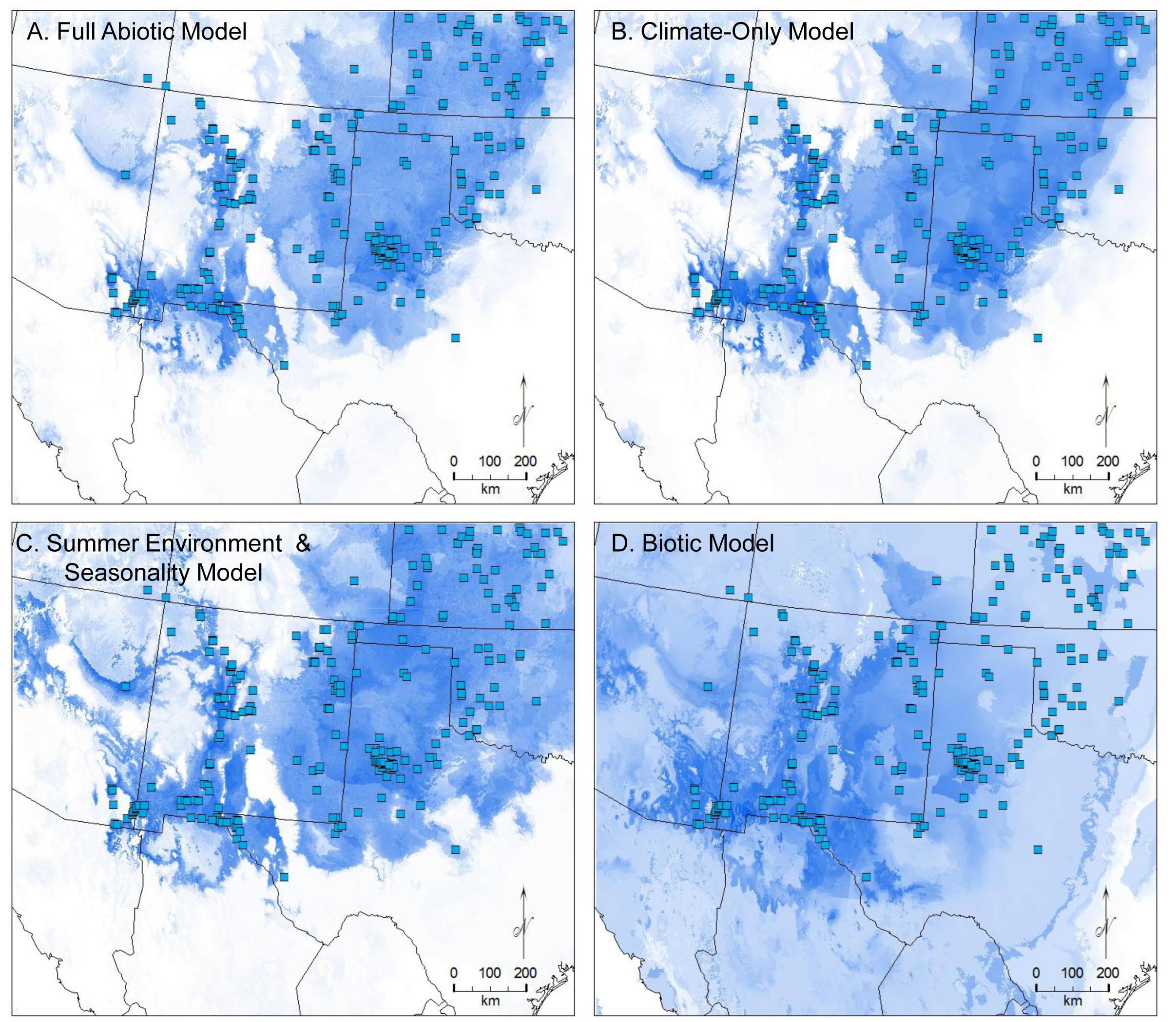

Supplement: Figure S1 — Maxent models for S. bombifrons . Map of S. bombifrons under all 4 models. Each pixel visually represents the logistic value for S. bombifrons for each of the 4 models, where values range from 0 (shown in white) to 1 (shown in dark blue). The four models are: A) the Full Abiotic Model, B) the Climate-Only Model, C) the Summer Environment & Seasonality Model, and D) the Biotic Model. (TIF) [file pone.0032748.s004.tif]

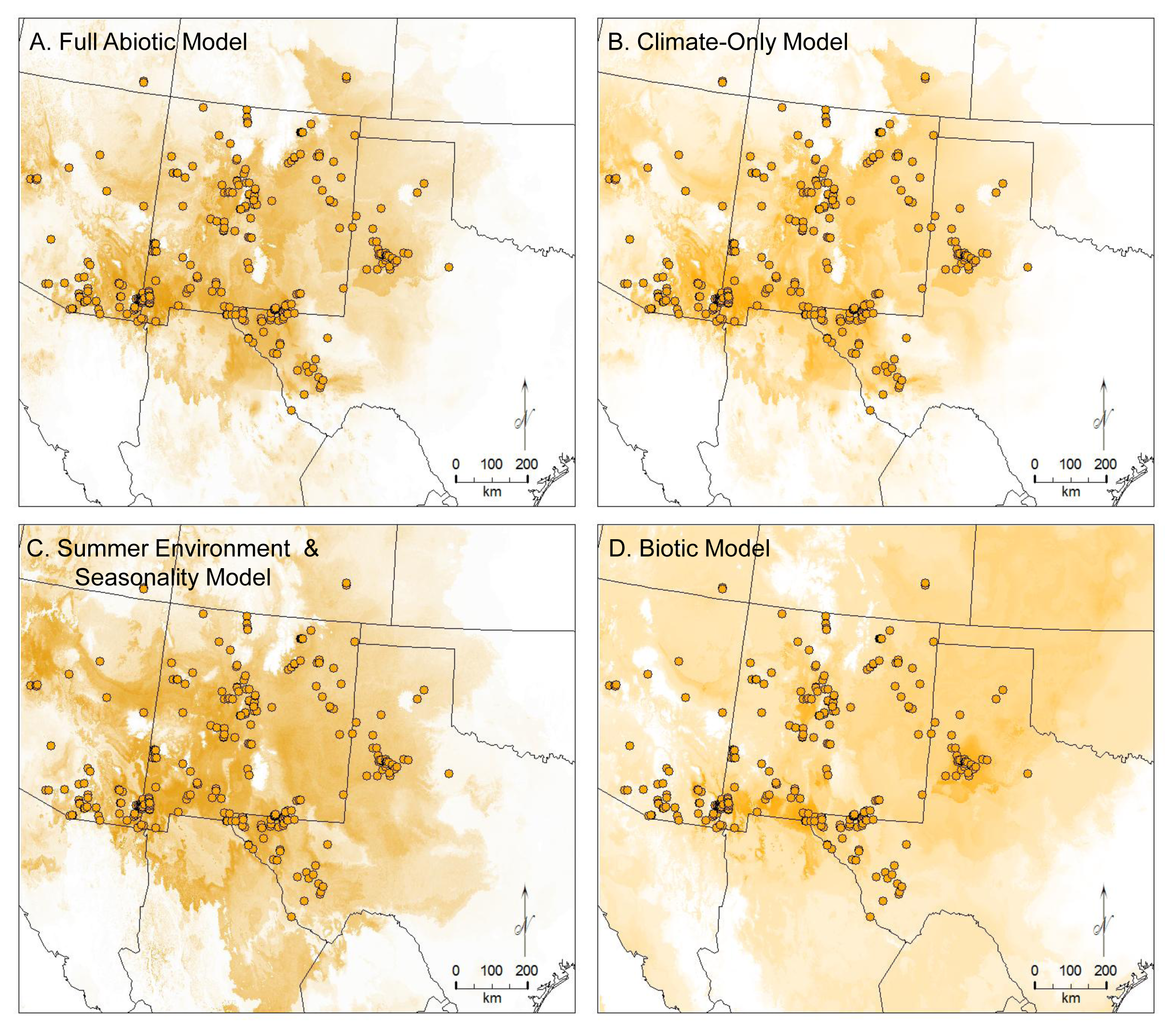

Supplement: Figure S2 — Maxent models for S. multiplicata . Map of S. multiplicata under all 4 models. Each pixel visually represents the logistic value for S. multiplicata for each of the 4 models, where values range from 0 (shown in white) to 1 (shown in dark orange). The four models are: A) the Full Abiotic Model, B) the Climate-Only Model, C) the Summer Environment & Seasonality Model, and D) the Biotic Model. (TIF) [file pone.0032748.s005.tif]

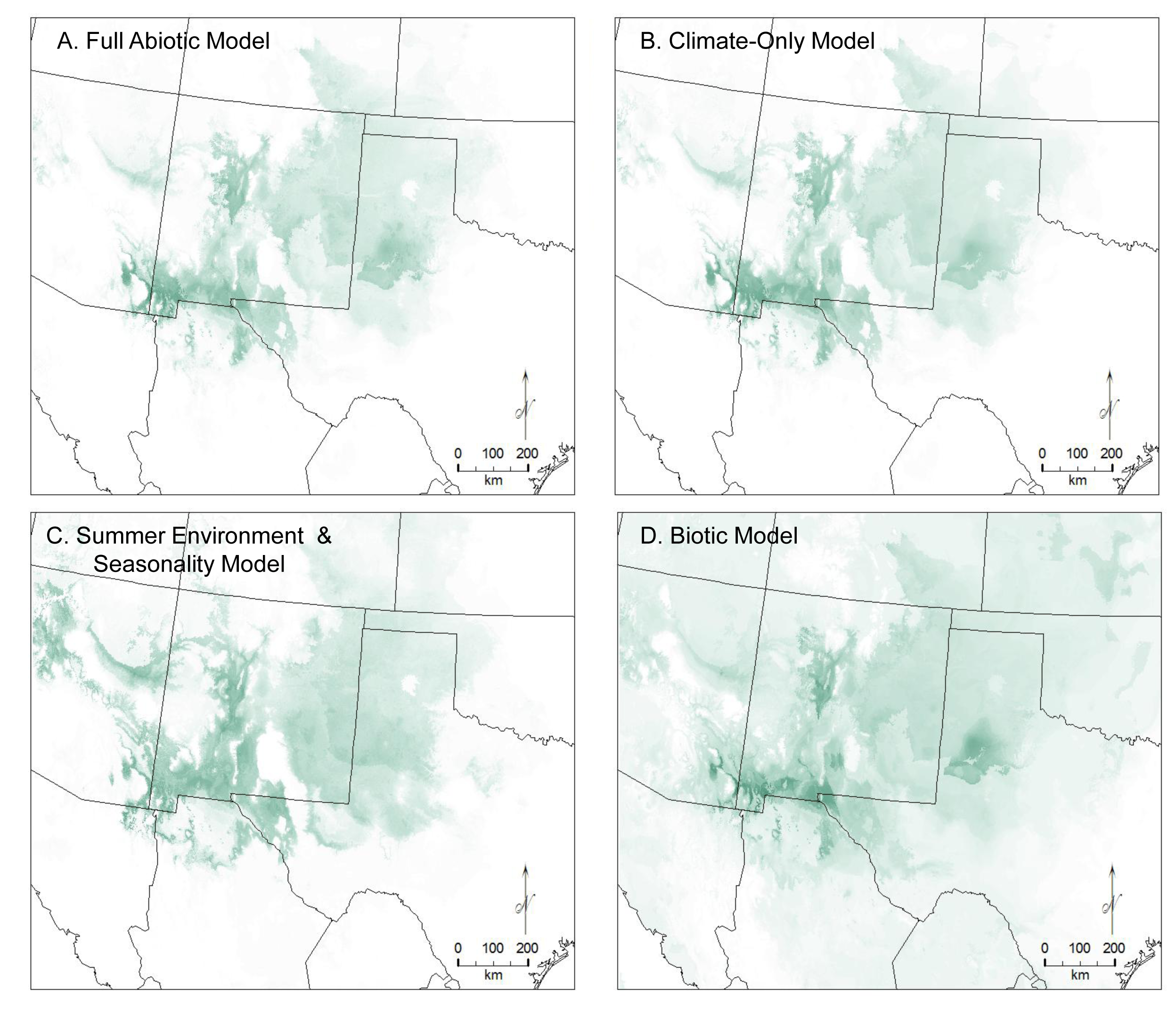

Supplement: Figure S3 — Maxent models of sympatry. Map of predicted sympatry under all 4 models. The value for each pixel was calculated by multiplying the logistic value of both species for each model, and values range from 0 (shown in white) to 1 (shown in dark green). The four models are: A) the Full Abiotic Model, B) the Climate-Only Model, C) the Summer Environment & Seasonality Model, and D) the Biotic Model. (TIF) [file pone.0032748.s006.tif]

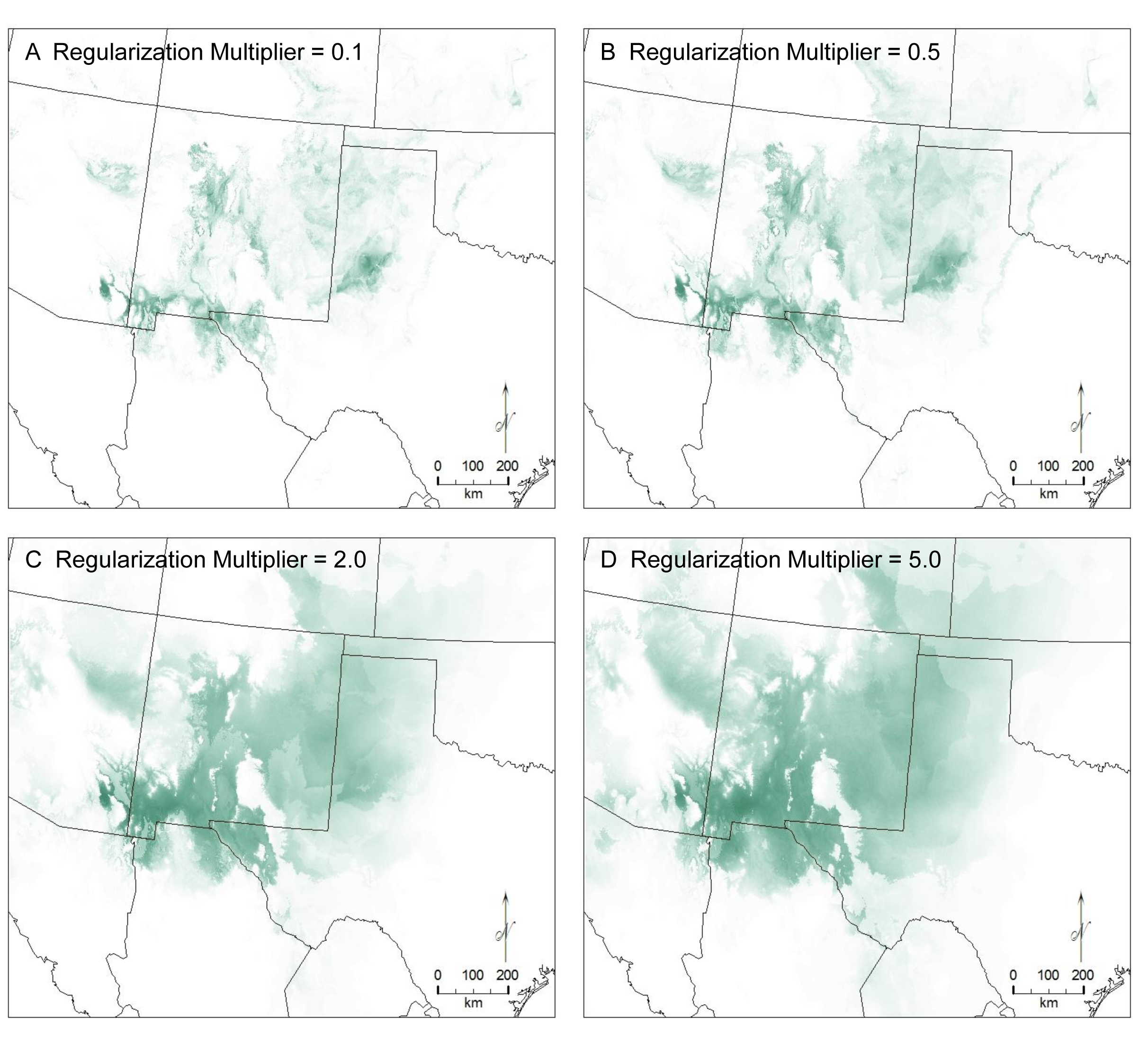

Supplement: Figure S4 — Sensitivity analysis. Maxent model results at four different regularization multipliers: A) 0.1, B) 0.5, C) 2.0, and D) 5.0. Each of these models was run based on the same environmental variables included in the Climate-Only model. Other than the regularization multiplier, all other values were held at default levels. Each map shows the average of 10 replicate runs. (TIF) [file pone.0032748.s007.tif]
