# Supplementary material for: Why Do Species Co-Occur? A Test of Alternative Hypotheses Describing Abiotic Differences in Sympatry versus Allopatry Using Spadefoot Toads
Source: PLoS One. 2012 Mar 30;7(3):e32748. doi: 10.1371/journal.pone.0032748 (PMC3316550; doi:10.1371/journal.pone.0032748)
Supplement: Table S1 — The following institutions provided locality data used in the ecological niche models. (DOCX) [file pone.0032748.s008.docx]

| **Table S1**. The following institutions provided locality data used in the ecological niche models: |
| --- |
| Amphibian and Reptile Collection, University of Arizona |
| Arizona State University |
| Auburn University Natural History Museum |
| California Academy of Sciences |
| Carnegie Museum of Natural History |
| Centennial Museum, University of Texas at El Paso |
| Cornell University |
| Division of Vertebrate Natural History, Michigan State University Museum |
| Field Museum of Natural History |
| Florida Museum of Natural History |
| Illinois Natural History Survey, University of Illinois |
| Indiana State University Vertebrate Collection |
| Louisiana Museum of Natural History, Louisiana State University |
| Milwaukee Public Museum |
| Museum of Comparative Zoology, Harvard University |
| Museum of Southwestern Biology, University of New Mexico |
| Museum of Vertebrate Zoology, University of California Berkeley |
| Natural History Museum of Los Angeles County |
| Nebraska State Museum |
| Peabody Museum, Yale University |
| Sam Noble Oklahoma Museum, University of Oklahoma |
| Smithsonian National Museum of Natural History |
| Texas Cooperative Wildlife Collection, Texas A&M University |
| Texas Natural History Center, University of Texas, Austin |
| University of Alabama Museum of Natural History |
| University of Colorado Museum |
| University of Illinois Museum of Natural History |
| University of Kansas Natural History Museum and Biodiversity Research Center |
| University of Louisiana at Monroe |
