# Supplementary material for: Why Do Species Co-Occur? A Test of Alternative Hypotheses Describing Abiotic Differences in Sympatry versus Allopatry Using Spadefoot Toads
Source: PLoS One. 2012 Mar 30;7(3):e32748. doi: 10.1371/journal.pone.0032748 (PMC3316550; doi:10.1371/journal.pone.0032748)
Supplement: Table S2 — Environmental variables used in each Maxent model. (DOCX) [file pone.0032748.s009.docx]

**Table S2.** Environmental variables used in each Maxent model.

|  |  |  |  |  |  |
| --- | --- | --- | --- | --- | --- |
| Source | Variable | Used in Model | | | |
|  |  | Full | Climate | Summer | Biotic |
|  | Temperature Annual Range | x | x | x |  |
|  | Mean Temperature of Coldest Quarter | x | x |  |  |
|  | Mean Temperature of Driest Quarter | x | x |  |  |
| WorldClim | Mean Diurnal Range in Temperature | x | x |  |  |
|  | Maximum Temperature of Warmest Month | x | x | x |  |
|  | Precipitation of Driest Quarter | x | x |  |  |
|  | Precipitation of Warmest Quarter | x | x | x |  |
|  | Precipitation of Coldest Quarter | x | x |  |  |
| USGS | Compound Topographic Index (i.e. Wetness Index) | x |  | x |  |
|  | Slope | x |  | x |  |
| Maxent Output | Logistic output for *S. multiplicata* |  |  |  | x |
|  | Logistic output for *S. bombifrons* |  |  |  | x |
|  |  |  |  |  |  |
|  |  |  |  |  |  |

The Full Abiotic Model uses all abiotic layers; the Climate-Only Model uses only climate layers; the Summer Environment and Seasonality Model uses climate layers from warm, wet periods in addition to hydrology and seasonality measures; and the Biotic Model uses the logistic output of the other species from the Climate-Only Model (i.e. the best performing abiotic model).
