# Supplementary material for: Why Do Species Co-Occur? A Test of Alternative Hypotheses Describing Abiotic Differences in Sympatry versus Allopatry Using Spadefoot Toads
Source: PLoS One. 2012 Mar 30;7(3):e32748. doi: 10.1371/journal.pone.0032748 (PMC3316550; doi:10.1371/journal.pone.0032748)
Supplement: Table S3 — Mean and standard deviation for each of the four niche models run. (DOCX) [file pone.0032748.s010.docx]

**Table S3**. Mean and standard deviation for each of the four niche models run.

|  | *S. bombifrons* | | *S. multiplicata* | |
| --- | --- | --- | --- | --- |
| Model | mean AUC | standard deviation | mean AUC | standard deviation |
| Full | 0.853 | 0.032 | 0.877 | 0.030 |
| Climate-only | 0.862 | 0.031 | 0.881 | 0.018 |
| Summer | 0.830 | 0.026 | 0.846 | 0.030 |
| Biotic | 0.736 | 0.051 | 0.747 | 0.037 |

The four models run are: the Full Abiotic Model, the Climate-only Model, the Summer Environment and Seasonality Model, and the Biotic model. All values are based on the average map produced from ten replicate runs of each model.
