# Supplementary material for: Why Do Species Co-Occur? A Test of Alternative Hypotheses Describing Abiotic Differences in Sympatry versus Allopatry Using Spadefoot Toads
Source: PLoS One. 2012 Mar 30;7(3):e32748. doi: 10.1371/journal.pone.0032748 (PMC3316550; doi:10.1371/journal.pone.0032748)
Supplement: Table S4 — Mean and standard deviation for each of the four niche models run for the sensitivity analysis. (DOCX) [file pone.0032748.s011.docx]

**Table S4**. Mean and standard deviation for each of the four niche models run for the sensitivity analysis.

|  | *S. bombifrons* | | *S. mulitplicata* | |
| --- | --- | --- | --- | --- |
| Regularization multiplier | mean AUC | standard deviation | mean AUC | standard deviation |
| 0.1 | 0.861 | 0.030 | 0.877 | 0.034 |
| 0.5 | 0.867 | 0.032 | 0.878 | 0.036 |
| 2.0 | 0.852 | 0.027 | 0.875 | 0.030 |
| 5.0 | 0.824 | 0.022 | 0.858 | 0.027 |

The environmental variables and point data were identical to the Climate-Only model above. Here, the regularization multiplier varied from 0.1 to 5.0.
